# Loading of Primary Human T Lymphocytes with Citrate-Coated Superparamagnetic Iron Oxide Nanoparticles Does Not Impair Their Activation after Polyclonal Stimulation

**DOI:** 10.3390/cells9020342

**Published:** 2020-02-01

**Authors:** Marina Mühlberger, Harald Unterweger, Julia Band, Christian Lehmann, Lukas Heger, Diana Dudziak, Christoph Alexiou, Geoffrey Lee, Christina Janko

**Affiliations:** 1 Department of Otorhinolaryngology, Head and Neck Surgery, Section of Experimental Oncology and Nanomedicine (SEON), Else Kröner-Fresenius-Stiftung-Professorship, Universitätsklinikum Erlangen, 91054 Erlangen, Germany; marina.muehlberger@uk-erlangen.de (M.M.);; 2 Department of Chemistry and Pharmacy, Division of Pharmaceutics, Friedrich-Alexander-Universität Erlangen-Nürnberg, 91058 Erlangen, Germany; 3 Department of Dermatology, Laboratory of Dendritic Cell Biology, Universitätsklinikum Erlangen, 91052 Erlangen, Germany; 4 Medical Immunology Campus Erlangen (MICE), Friedrich-Alexander-Universität Erlangen-Nürnberg, 91054 Erlangen, Germany

**Keywords:** SPION, T cells, nanomedicine, cancer, solid tumor, magnetic targeting, T cell activation, immune therapy

## Abstract

For the conversion of immunologically cold tumors, characterized by a low T cell infiltration, into hot tumors, it is necessary to enrich T cells in the tumor area. One possibility is the use of magnetic fields to direct T cells into the tumor. For this purpose, primary T cells that were freshly isolated from human whole blood were loaded with citrate-coated superparamagnetic iron oxide nanoparticles (SPION^Citrate^). Cell toxicity and particle uptake were investigated by flow cytometry and atomic emission spectroscopy. The optimum loading of the T cells without any major effect on their viability was achieved with a particle concentration of 75 µg Fe/mL and a loading period of 24 h. The cellular content of SPION^Citrate^ was sufficient to attract these T cells with a magnet which was monitored by live-cell imaging. The functionality of the T cells was only slightly influenced by SPION^Citrate^, as demonstrated by in vitro stimulation assays. The proliferation rate as well as the expression of co-stimulatory and inhibitory surface molecules (programmed cell death 1 (PD-1), lymphocyte activation gene 3 (LAG-3), T cell immunoglobulin and mucin domain containing 3 (Tim-3), C-C motif chemokine receptor 7 (CCR7), CD25, CD45RO, CD69) was investigated and found to be unchanged. Our results presented here demonstrate the feasibility of loading primary human T lymphocytes with superparamagnetic iron oxide nanoparticles without influencing their viability and functionality while achieving sufficient magnetizability for magnetically controlled targeting. Thus, the results provide a strong fundament for the transfer to tumor models and ultimately for new immunotherapeutic approaches for cancer treatment.

## 1. Introduction

Despite the improvement of cancer therapy in the past years, in many cases, cancer has remained an incurable disease [1]. In the last decades, it has become clear that the immunological tumor environment is crucial for the efficacy of many, if not all cancer therapies [2,3,4,5]. This is demonstrated by the strong prognostic value of tumor infiltrating lymphocytes (TILs) for the patient’s clinical outcome [6,7,8]. Concerning the infiltration of solid tumors by immune cells, these tumors can by categorized into so-called “hot” and “cold” tumors. Whereas an immunological “hot” tumor is characterized by a strong T cell infiltration, in “cold” tumors, no T cells are found, or they are only localized in the tumor periphery [9]. If a patient has a “hot” tumor, the prognosis and chances for a cure are much higher. Many of these patients may profit from immunostimulatory therapies, such as checkpoint inhibitors, e.g., nivolumab or ipilimumab. These checkpoint inhibitors block the interaction of inhibitory receptors on the T cells with the corresponding ligands, thereby releasing the brakes of the immune system allowing for a stronger attack of the tumor [10,11]. Despite the potency of checkpoint inhibitors, for many tumor types, only a small fraction of patients profit from the therapy in the long run, which might be associated with the immunological status of their tumor and metastases (“cold” versus “hot”) [12]. Therefore, we aim to foster the migration of T cells into tumors by turning them into magnetically guidable cells by loading with superparamagnetic iron oxide nanoparticles (SPIONs). This allows for their control and enrichment within the tumor by the application of an external magnetic field. Thereby, an immunological “cold” tumor can be transformed into a “hot” tumor and the patient may profit from a better prognosis and a higher efficacy of checkpoint inhibitors.

Other research groups apply different strategies to attract cytotoxic effector cells, such as T or natural killer (NK) cells, to tumor cells. One example is bi-specific T cell engagers (BiTEs). These antibodies have one specificity for the tumor cells and one for the effector cells, thereby bridging both to enable killing of the tumor cells [13]. This strategy is efficient but also limited to non-solid and selected solid tumors as these BiTEs only have very short plasma half-lives and therefore may not penetrate enough into solid tumors [14,15]. Furthermore, the effector cells needed might not be available in sufficient numbers in “cold” tumors. Additionally, BiTE antibodies may induce a strong cytokine release syndrome as these antibodies can activate the effector T and/or NK cells in an antigen-independent and therefore polyclonal fashion [16,17,18]. In contrast, the magnetic labelling of T cells may be used for many different solid tumors and can be extended to other cell types, such as NK cells, dendritic cells, stem cells or even chimeric antigen receptor (CAR)-T cells, in order to reduce potentially detrimental side effects [19,20,21,22,23].

In previous studies, we have demonstrated the feasibility of the loading of murine lymphoma T cell lines with SPIONs. This process allowed for the attraction of the loaded T cells with a magnetic field after purification from excess SPIONs [24]. Also, Sanz-Ortega et al. recently demonstrated the possibility of magnetically enriching T cells loaded with variously coated SPIONs [25]. Due to their low cytotoxicity, citrate-coated SPIONs (SPION^Citrate^) have proven to be suitable for this application [26].

In this study, we aimed to transfer the established methods for loading of immortalized T cells to freshly isolated human blood CD3^+^ T cells. We determined an optimal dose and incubation time for the loading of the primary T cells, minimizing cytotoxic effects while maximizing particle uptake and thus their magnetizability. To gain insights into the functionality of the loaded T cells, we investigated their behavior in in vitro T cell stimulation assays. We compared non-loaded T cells with SPION^Citrate^-loaded cells concerning their proliferation, interleukin-2 (IL-2) secretion as well as their expression for several activation markers and checkpoint molecules.

## 2. Materials and Methods

### 2.1. Materials

Nitric acid 65%, formic acid 98%, sodium carbonate, sodium chloride, sodium sulfate, formaldehyde solution 37%, Triton X-100, Tween 20, ammonia solution 25% and acetone were supplied by Carl Roth (Karlsruhe, Germany), and sodium citrate dihydrate, iron (II) chloride tetrahydrate, iron (III) chloride hexahydrate and Muse Count and Viability Kit were supplied by Merck (Darmstadt, Germany). S-Monovettes 10 mL 9NC, S-Monovettes 2.7 mL K3E and two-well tissue culture chambers on glass (detachable) were purchased from Sarstedt (Nuembrecht, Germany), Minisart NML syringe filters (0.2 µm pore size) from Sartorius (Goettingen, Germany) and Falcon cell strainers 40 µm from Corning Life Sciences (Corning, NY; USA). Ringer’s solution was obtained from Fresenius Kabi (Bad Homburg, Germany), recombinant human IL-2 from ImmunoTools (Friesoythe, Germany) and ImmunoCult Human CD3/CD28/CD2 T Cell Activator from Stemcell Technologies (Vancouver, BC, Canada). Penicillin streptomycin-solution 5000 U/mL, GlutaMAX supplement, 4′,6-diamidino-2-phenylindole dihydrochloride (DAPI), Hoechst 33342 (Hoe), Annexin A5 fluorescein isothiocyanate (FITC) conjugate (AxV), DiIC_1_(5) (1,1′-dimethyl-3,3,3′,3′-tetramethylindodicarbocyanine iodide, DiI), Nunc MaxiSorp flat-bottom 96-well ELISA plates, eBioscience PerCP-Cy5.5 CD25 monoclonal antibody (clone BC96), eBioscience PerCP-Cy5.5 mouse IgG1, κ isotype control (clone P3.6.2.8.1) and gibco RPMI medium 1640 (without L-glutamine) were supplied by Thermo Fisher Scientific (Waltham, MA, USA), phosphate-buffered saline (PBS), human serum from human male AB plasma, propidium iodide (PI) and Lucifer Yellow CH dipotassium salt (LY) by Sigma-Aldrich (Taufkirchen, Germany) and fetal calf serum (FCS) and amphotericin B (250 µg/mL) by Biochrom (Berlin, Germany). The block magnet Q-19-13-06-LN was obtained from supermagnete Webcraft (Gottmadingen, Germany), human CD3 Fab-TACS Gravity Kit from IBA (Goettingen, Germany). BD Pharmingen FITC mouse anti-human CD3 antibody (clone UCHT1), BD Pharmingen FITC mouse IgG1, κ isotype control (clone MOPC-21), BD Pharmingen PE-Cy7 mouse anti-human CD69 antibody (clone FN50), BD Horizon BUV 395 mouse anti-human CD3 antibody (clone UCHT1) and BD Horizon BUV 737 mouse anti-human CD8 antibody (clone SK1) were provided by BD Biosciences (San Jose, CA, USA). CFSE cell division tracker kit, ELISA Max Standard human IL-2 Set, plate sealers, coating buffer, TMB substrate set, stop solution, PerCP-Cy5.5 anti-human CD4 antibody (clone OKT4), PerCP-Cy5.5 mouse IgG2b, κ isotyope control (clone MPC-11), APC anti-human CD8 antibody (clone HIT8a), APC mouse IgG1, κ isotype control (clone MOPC-21), Pacific Blue anti-human CD8 antibody (clone HIT8a), Pacific Blue mouse IgG1, κ isotype control (clone MOPC-21), PE anti-human CD197 (CCR7) antibody (clone G043H7), PE mouse IgG2a, κ isotype control (clone MOPC-173),) APC anti-human CD45RO antibody (clone UCHL1), APC mouse IgG2a, κ isotype control (clone MOPC-173), Brilliant Violet 421 mouse IgG2a, κ isotype control (clone MOPC-173), Brilliant Violet 650 anti-human CD279 (PD-1) antibody (clone EH12.2H7), Brilliant Violet 650 mouse IgG1, κ isotype control (clone MOPC-21), Brilliant Violet 711 anti-human CD223 (LAG-3) antibody (clone 11C3C65), Brilliant Violet 711 mouse IgG1, κ isotype control (clone MOPC-21), PE/Dazzle594 anti-human CD366 (Tim-3) antibody (clone F38-2E2), PE/Dazzle594 mouse IgG1, κ isotype control (clone MOPC-21), PE anti-human CD4 antibody (clone RPA-T4) and PE-Cy7 mouse IgG1, κ isotype control (clone MOPC-21) were purchased from BioLegend (San Diego, CA, USA). The water used for all experiments came from a Merck Milli-Q Direct water purification system (Darmstadt, Germany).

### 2.2. Nanoparticle Synthesis

As recently published, SPION^Citrate^ were synthesized according to a modified protocol of Elbialy et al. [26,27]. Briefly, under a protective argon atmosphere, iron(II) chloride and iron(III) chloride in a ratio of 1:2 were dissolved in water and stirred. Precipitation of iron oxide was achieved by adding ammonia solution 25%. After the addition of 1 M sodium citrate solution, the mixture was stirred for 30 min at 90 °C. SPIONs were allowed to cool down to room temperature before removing excess sodium citrate by washing with acetone five times. The nanoparticles were dried under vacuum and stored in a desiccator. For characterization and usage, the particles were dispersed in the required iron concentration in the appropriate amount of water and sterilized by means of a 0.2 µm pore size syringe filter.

### 2.3. Nanoparticle Characterization

To characterize SPION^Citrate^ after sterile filtration, their iron content, hydrodynamic size, zeta potential, magnetic susceptibility and the stability of the particles in human whole blood were investigated.

After dissolving the samples in nitric acid 65%, they were diluted with water and the iron concentration was measured using atomic emission spectroscopy (AES) with an Agilent 4200 MP-AES (Agilent Technologies, Santa Clara, CA, USA). Commercially available iron solution (1000 mg/L, Bernd Kraft, Duisburg, Germany) was used as external standard.

The particles‘ hydrodynamic size at 25 °C in water and in RPMI 1640 cell culture medium (supplemented with 10% heat-inactivated fetal calf serum (HI-FCS), 1% l-glutamine, 2% penicillin streptomycin-solution and 1% amphotericin B) was examined at an iron concentration of 50 µg/mL by dynamic light scattering (DLS) with a Zetasizer Nano ZS (Malvern Panalytical, Almelo, Netherlands). The following parameters were applied for the measurements: water refractive index 1.33, viscosity 0.8872 mPa·s, supplemented RPMI 1640 cell culture medium refractive index 1.335, viscosity 1.1100 mPa·s.

To check the aqueous zeta potential, SPION^Citrate^ was measured in triplicate at an iron concentration of 100 µg/mL and pH 6.6 with the Zetasizer Nano ZS. The dielectric constant was set to 78.5.

A MS2G magnetic susceptibility meter (Bartington Instruments, Oxfordshire, UK) was used to determine the magnetizability of SPION^Citrate^ at an iron concentration of 1 mg/mL.

The stability of the nanoparticles in blood was tested according to a protocol published by Lugert et al. [28]. SPION^Citrate^ were incubated for 45 min in freshly drawn human whole blood (stabilized against coagulation with citrate). Water served as a negative control. After incubation, samples were streaked on glass slides and analyzed with a Zeiss Axio Observer Z1 microscope (Carl Zeiss, Jena, Germany). The use of human material (blood) was approved by the local ethics committee at the Universitätsklinikum Erlangen (reference number 257_14 B) and written informed consent was obtained from donors.

### 2.4. Cell Isolation

The isolation of CD3^+^ T cells from freshly drawn, citrate-anticoagulated human blood was performed using the CD3 Fab-TACS Gravity Kit (IBA, Goettingen, Germany) according to the manufacturer’s instructions.

For all experiments, cells were cultured in RPMI 1640 medium supplemented with 10% HI-FCS, 1% l-glutamine, 2% penicillin streptomycin-solution and 1% amphotericin B at 37 °C in a humidified 5% CO_2_ atmosphere.

T cells were checked after isolation for changes in the CD4^+^ and CD8^+^ ratio due to the treatment. In parallel to cell isolation, whole blood was processed by lyzing the erythrocytes with formic acid 0.12% (pH 2.7) [29]. After neutralization with alkaline buffer solution (Na_2_CO_3_ 6 g/L, NaCl 14.5 g/L, Na_2_SO_4_ 31.1 g/L in water, pH 11.2) and washing, the pellet of the remaining blood cells was resuspended in medium.

The whole blood cells (after lysis of erythrocytes) and the isolated T cells were incubated with fluorescein isothiocyanate (FITC) anti-human CD3, peridinin-chlorophyll-protein complex-cyanine 5.5 (PerCP-Cy5.5) anti-human CD4 and allophycocyanin (APC) anti-human CD8 antibodies or with their isotype controls, FITC mouse immunoglobulin G (IgG) 1 κ, PerCP-Cy5.5 mouse IgG2b κ and APC mouse IgG1 κ, respectively. The experiment was performed in triplicate for 30 min at 4 °C under light protection. The cells were then washed with phosphate-buffered saline (PBS) and resuspended and fixed in 1% formaldehyde in PBS. Fluorescence was measured with a Gallios flow cytometer and analyzed with Kaluza 1.2 Analysis Software (Beckman Coulter Life Sciences, Indianapolis, IN, USA).

The results are provided within the Supplementary Information (Table S1 and Figure S1).

### 2.5. Cell Toxicity

Potential toxic effects of SPION^Citrate^ were examined using flow cytometry [30]. 2.5 × 10^5^ T cells in 1 mL medium were seeded in triplicate in 24-well plates. They were incubated with SPION^Citrate^ at an iron concentration of 25, 50, 75 or 100 µg/mL or the same volume of water as control. A sample of 50 µL was taken from each well immediately after mixing cells with SPIONs, as well as after 24 and 48 h. Cells were stained with a mixture containing 1 µL/mL Hoechst 33342 (Hoe, 10 mg/mL), 2 µL/mL Annexin A5 FITC conjugate (AxV), 66.7 ng/mL propidium iodide (PI) and 0.4 µL/mL DiIC_1_(5) (1,1′-dimethyl-3,3,3′,3′-tetramethylindodicarbocyanine iodide, DiI, 10 µM) in Ringer’s solution. After incubation at 4 °C under light protection for 20 min, fluorescence was measured with a Gallios flow cytometer and analyzed with Kaluza 1.2 Analysis Software. For this, Hoe was used to identify cells by their nuclei. With AxV and PI, apoptotic and necrotic cells were detected. Additionally, DiI was used to determine mitochondrial membrane potential integrity and thus confirmed the gating of viable cells in the AxV/PI analysis.

### 2.6. Cellular Uptake

To obtain information about the particle uptake into the T cells, analysis of the cell granularity, staining with the fluorescent dye Lucifer Yellow (LY) and spectroscopical quantification of the cellular iron content were combined.

For staining of cells with LY, 2.5 × 10^5^ cells in 1 mL medium containing 2 µg LY were seeded in triplicate in 24-well plates. They were incubated with SPION^Citrate^ at an iron concentration of 25, 50, 75 or 100 µg/mL or the same volume water as control. A sample of 50 µL was taken from each well immediately after mixing cells with SPIONs, as well as after 24 and 48 h. Cells were stained with a mixture containing 1 µL/mL Hoe (10 mg/mL), 66.7 ng/mL PI and 0.4 µL/mL DiI (10 µM) in Ringer’s solution. After incubation at 4 °C under light protection for 20 min, fluorescence was measured with a Gallios flow cytometer and analyzed with Kaluza 1.2 Analysis Software.

The cell granularity was analyzed from the LY flow cytometry data. The side scatter (SSc) values of viable cells were compared as the internalization or adhesion of nanoparticles to the cells alters the cell granularity [31].

To quantify the uptake of SPIONs into the T cells, 7.5 × 10^6^ cells in 20 mL medium were incubated for 24 h with SPION^Citrate^ at an iron concentration of 75 or 100 µg/mL or the same volume of water as control. After incubation, cells were washed with PBS to remove excess SPIONs. The washed cells were resuspended in medium and their cell count was determined by a MUSE Cell Analyzer. The volume corresponding to 5 × 10^5^ cells was taken from each sample in triplicate. Cell pellets were formed by centrifugation at 300 rcf for 5 min and were washed with PBS. The pellets were dried at 95 °C for 30 min and lysed with nitric acid at 95 °C. The samples were diluted with water and their iron content was measured by AES with an Agilent 4200 MP-AES. A commercially available iron solution with 1000 mg Fe/L was used as an external standard.

### 2.7. Magnetizability

The attraction of SPION^Citrate^-loaded T cells by a magnet was tested while filming under a microscope. 7.5 × 10^6^ cells in 20 mL medium were incubated for 24 h with or without SPION^Citrate^ at an iron concentration of 75 µg/mL. After incubation, cells were washed with PBS to remove excess SPIONs. The washed cells were resuspended in medium and their cell count was determined by a MUSE Cell Analyzer. 1 × 10^6^ cells of each sample were transferred to one well of a two-well chamber on a glass slide at a total volume of 1 mL. Under an Axio Observer Z.1 microscope, a lengthwise magnetized block magnet was placed in the second well and the cells were filmed at the edge of the barrier between the two wells.

### 2.8. T Cell Proliferation and Cell Cycle

3 × 10^7^ cells were incubated for 24 h at 37 °C with or without 75 µg Fe/mL SPION^Citrate^. The cells were then washed with fresh medium and resuspended in 1.5 mL PBS supplemented with 5% HI-FCS. Cell count was determined using the MUSE Cell Analyzer throughout the experiment. A third part of each loaded and non-loaded cells was diluted with medium to a concentration of 1 × 10^6^ cells/mL and incubated at 37 °C for 24 h. The remaining two thirds of the cells in each 1 mL PBS + 5% HI-FCS were stained with carboxyfluorescein succinimidyl ester (CFSE) after a modified protocol published by Quah et al. [32]. In brief, cells were stained with 5 µM carboxyfluorescein diacetate succinimidyl ester (CFDA SE) for 5 min at room temperature under light protection. The staining process was stopped by adding 20 mL medium and cells were washed twice with PBS + 5% HI-FCS. Stained loaded and non-loaded cells were each diluted to a concentration of 1·10^6^ cells/mL and incubated at 37 °C in the dark overnight. The next day, the medium of the cells was replaced by 5 mL fresh medium each and the cell count was determined. Each sample was diluted with medium to a concentration of 2 × 10^5^ cells/mL and 600 µL per well were seeded in 48-well plates in triplicate per measurement time (t = 0, 24, 48, 72, 96 and 120 h after stimulation). For the t = 0 h values, 100 µL aliquots were stained for 20 min at 4 °C under light protection with 100 µL PBS containing 166.7 ng/mL PI and subsequently analyzed in Gallios flow cytometer.

To stimulate the T cells, 12.5 µL Immunocult human CD3/CD28/CD2 T cell activator and 30 IU recombinant human IL-2 (rh IL-2) per 1 mL were added to the cell suspension. The stimulated cells were seeded in the same way as the unstimulated cells. After t = 24, 48 and 72 h, 100 µL aliquots were stained with 100 µL PBS containing 166.7 ng/mL PI and analyzed in flow cytometry. After 72 h, 600 µL medium with (stimulated cells) or without (unstimulated cells) 60 IU/mL rh IL-2 were added per well. After t = 96 and 120 h, 200 µL aliquots were stained with 100 µL PBS containing 166.7 ng/mL PI and analyzed in flow cytometry.

The DNA content of the cells was measured during the experiment according to a modified protocol published by Riccardi et al. [33]. Only the unstained cells were used and a further 100 µL (t = 0, 24, 48 and 72 h after stimulation) or 200 µL (t = 96 and 120 h after stimulation) sample per well was taken at each CFSE measurement time. To extract and stain the DNA of the cells, 500 µL staining solution were added to each sample and incubated over night at 4 °C under light protection. The staining solution consisted of 1 g/L trisodium citrate dihydrate, 1 mL/L Triton X-100 and 1 mg/L PI in water. Fluorescence was measured with a Gallios flow cytometer and analyzed with Kaluza 1.2 Analysis Software.

### 2.9. T Cell Subsets and Activation Markers

For phenotyping of different T cell subsets, 2 × 10^7^ cells were incubated for 24 h at 37 °C with or without 75 µg Fe/mL SPION^Citrate^. After 24 h, cells were washed and resuspended in 5 mL fresh medium. Cell count was determined using the MUSE Cell Analyzer. Cells were each diluted with medium to a concentration of 2 × 10^5^ cells/mL. 600 µL per well of the unstimulated cells were seeded in 48-well plates in triplicate per time (t = 0, 72, 120 h after stimulation).

For t = 0 h, 100 µL were taken from each of the three wells and transferred in 5 mL tubes. Cells were stained with anti-CD3, anti-CD4 and anti-CD8 antibodies as well as with anti-CD197 (C-C motif chemokine receptor 7 (CCR7)) and anti-CD45RO antibodies or respective isotype controls (Table 1). After incubation at 4 °C for 30 min, samples were washed with PBS, fixed in 250 µL formaldehyde 1% in PBS and subsequently analyzed in the Gallios flow cytometer.

To stimulate the T cells, 12.5 µL Immunocult human CD3/CD28/CD2 T cell activator and 30 IU rh IL-2 per 1 mL were added to the cell suspension. The stimulated cells were seeded in the same way as the unstimulated cells. After t = 72 h, 100 µL were taken from each of stimulated and unstimulated cells and were transferred into 5 mL tubes, stained and measured as described above. Furthermore, 600 µL medium with (stimulated cells) or without (unstimulated cells) 60 IU/mL rh IL-2 were added to the wells. After t = 120 h, 200 µL were taken, transferred into 5 mL tubes, stained and measured as described above.

The expression of the markers programmed cell death 1 (PD-1), lymphocyte activation gene 3 (LAG-3), T cell immunoglobulin and mucin domain containing 3 (Tim-3), CD25 and CD69 was analyzed in a separate experiment. 2 × 10^7^ cells were loaded with nanoparticles as described above. The next day, cells were washed and resuspended in 10 mL fresh medium. Cell count was determined using the MUSE Cell Analyzer. Cells were each diluted with medium to a concentration of 2 × 10^5^ cells/mL and for unstimulated cells, 600 µL were seeded in triplicate per time (t = 0, 72, 120 h after stimulation) in 48-well plates. For the t = 0 h values, 200 µL each were taken and split into two wells of a 96-well plate with a V-shaped bottom. Cells were washed with PBS with 2% human AB-serum (FACS buffer) and resuspended in staining solution containing FACS buffer with antibodies or isotype controls, as listed in Table 2. After 30 min of incubation under light protection at 4 °C, cells were washed three times with FACS buffer and resuspended in 300 µL FACS buffer containing 1:10,000 diluted 4′,6-diamidino-2-phenylindole (DAPI, 1 mg/mL). Fluorescence was measured with a BD LSRFortessa cell analyzer (Becton, Dickinson and Company, Franklin Lakes, NJ, USA).

To stimulate the T cells, 12.5 µL CD3/CD28/CD2 T cell activator and 30 IU rh IL-2 per 1 mL were added to the cell suspension. 600 µL per well of the stimulated cells were seeded in 48-well plates and incubated at 37 °C. This was done in triplicate per measurement time (t = 72 and 120 h after stimulation). After t = 72 h, 200 µL of each unstimulated and stimulated samples were split into two wells of a 96-well plate with V bottom and stained and measured as described above. Furthermore, 600 µL medium with (stimulated cells) or without (unstimulated cells) 60 IU/mL rh IL-2 was added per well. After t = 120 h, 400 µL each were taken, split into two 200 µL samples in 5 mL tubes, stained and analyzed.

### 2.10. IL-2 Release

For the quantification of the cells’ IL-2 release, 2 × 10^7^ cells were incubated for 24 h at 37 °C with or without 75 µg Fe/mL SPION^Citrate^. Cells were then washed and resuspended in 5 mL fresh medium. Cell count was determined using the MUSE Cell Analyzer. Cells were each diluted with medium to a concentration of 2 × 10^5^ cells/mL. 250 µL per well of the unstimulated cells were seeded in 96-well plates with a round bottom and incubated at 37 °C. This was done in triplicate per measurement time (t = 0, 24, 72 and 120 h after stimulation). To stimulate the T cells, 12.5 µL human CD3/CD28/CD2 T cell activator per 1 mL was added to the cell suspension and cells were seeded in the same way as unstimulated cells. After t = 72 h, 60 µL fresh medium were added to every well to maintain cell viability. At t = 0, 24, 72 and 120 h, the plates were centrifuged at 300 rcf for 5 min and 150 µL supernatant from each of the respective wells were harvested and stored at −80 °C.

After all samples had been collected, the IL-2 content was quantified with the ELISA MAX Standard Set human IL-2 from BioLegend (San Diego, CA, USA) according to the manufacturer’s instructions. In brief, unstimulated samples and stimulated t = 0 h samples were used undiluted. Stimulated t = 24 h samples were diluted 1:100, stimulated t = 72 h samples, 1:500. The stimulated t = 120 h samples were also diluted 1:500 which corresponded to a dilution of 1:620 due to the addition of medium during the experiment.

In addition to each T cell activation experiment, the cells were monitored in triplicate in 48-well plates with the IncuCyte S3 Live-Cell Analysis System (Sartorius, Göttingen, Germany). Only unstained cells were used for live imaging and pictures were taken every 24 h.

### 2.11. Statistical Analysis

**Figure 1:**
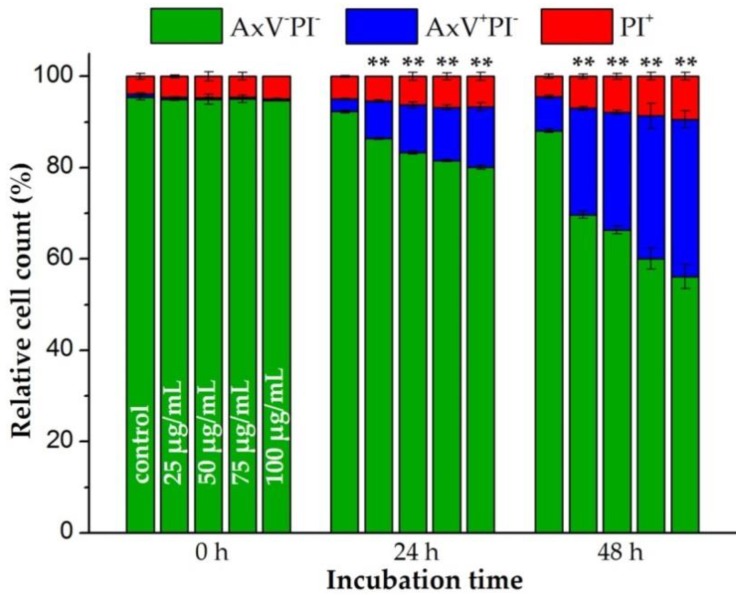
Effects of citrate-coated superparamagnetic iron oxide nanoparticles (SPION^Citrate^) on viability of human primary T lymphocytes. T cells were incubated with SPION^Citrate^ at selected iron concentrations (0–100 µg/mL). After 0, 24 and 48 h, cells were stained with Annexin A5 FITC conjugate (AxV) and propidium iodide (PI) to detect apoptotic and necrotic cells by flow cytometry. Viable cells (AxV^−^PI^−^) are displayed in green, apoptotic cells (AxV^+^PI^−^) in blue and necrotic cells (PI^+^) in red. The experiment was performed with three different donors. Shown are the mean values with standard deviations of one representative donor. Data of other donors can be found in Supplementary Figure S2. Significance for viable cells between treatment groups and control at the respective time is indicated by asterisks: (** *p* < 0.005). Abbreviations: AxV: Annexin A5 FITC conjugate, FITC: fluorescein isothiocyanate, PI: propidium iodide, SPION^Citrate^: citrate-coated superparamagnetic iron oxide nanoparticles.

**Figure 2:**
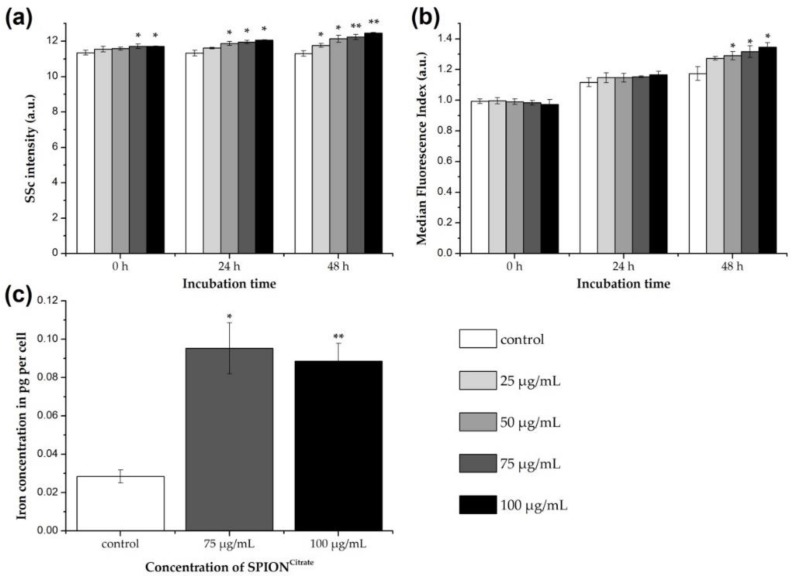
Association of citrate-coated superparamagnetic iron oxide nanoparticles (SPION^Citrate^) to human primary T lymphocytes. (**a**) Changes in cellular granularity due to superparamagnetic iron oxide nanoparticle (SPION) uptake or adhesion were determined by measuring the side scatter (SSc) in flow cytometry after incubation of T cells with SPION^Citrate^. Since cell death processes alter cellular morphology as well, we gated on viable DiIC_1_(5)^+^ (1,1′-dimethyl-3,3,3′,3′-tetramethylindodicarbocyanine iodide, DiI) cells with intact mitochondrial membrane potential. (**b**) T cells were incubated for 0, 24 and 48 h with SPION^Citrate^ (25–100 µg Fe/mL) and the membrane impermeable fluorescent dye Lucifer Yellow (LY). If SPIONs are taken up, LY gets co-ingested and serves therefore as indicator for intracellular SPION uptake. Since LY leaks from disrupted membranes, we gated on viable DiI^+^ cells with intact mitochondrial membrane potential. (**c**) Quantification of cellular iron content was performed by atomic emission spectroscopy after incubation of T cells with SPION^Citrate^ (75 and 100 µg Fe/mL) for 24 h. The experiments were performed with three different donors. Shown are the mean values with standard deviations of one representative donor. Data of other donors can be found in Supplementary Figure S3. Significance between treatment groups and control at the respective time is indicated by asterisks: * *p* < 0.05, ** *p* < 0.005. Abbreviations: DiI: DiIC_1_(5) (1,1′-dimethyl-3,3,3′,3′-tetramethylindodicarbocyanine iodide), LY: Lucifer Yellow, SPION: superparamagnetic iron oxide nanoparticle, SPION^Citrate^: citrate-coated superparamagnetic iron oxide nanoparticles, SSc: side scatter.

For Figures 1, 2, 4, 5 and 6, an unpaired Student‘s *t*-test was performed in MS Excel (Microsoft, Redmond, WA, USA). One-way analysis of variance (ANOVA) with the Bonferroni post-hoc test in Prism (ver. 5.01) was used in Figure 7. Asterisks mark statistical significance: * *p* < 0.05, ** *p* < 0.005.

## 3. Results and Discussion

### 3.1. Nanoparticle Characterization

As previously described, sterile filtered SPION^Citrate^ were characterized with regard to hydrodynamic diameter, zeta potential, magnetic susceptibility and blood stability [26].

SPION^Citrate^ had a mean hydrodynamic diameter of 51 nm (Z-Average) with a corresponding polydispersity index (PDI) of 0.132 in water and a mean diameter of 124 nm with a corresponding PDI of 0.250 in cell culture medium. The particles’ zeta potential in water was −41.9 ± 1.2 mV at pH 6.6. At an iron concentration of 1 mg/mL, the magnetic susceptibility was 4.08 × 10^−3^. SPION^Citrate^ did not show any signs of agglomeration in freshly drawn citrate-stabilized human whole blood.

### 3.2. SPION^Citrate^ Loading Does Not Have Major Effects on Primary Human T Cell Viability

The toxicity of SPION^Citrate^ had previously been tested up to an iron concentration of 100 µg/mL using the murine T cell line EL4 (ATCC TIB-39), showing a good biocompatibility [26]. To analyze the magnetic labelling of human primary T cells, we isolated CD3^+^ T cells from human whole blood (Supplementary Table S1, Figure S1). The same concentration range of 25 to 100 µg Fe/mL was investigated for isolated primary human T cells of at least three donors. T cells were incubated with SPION^Citrate^ for 0, 24 and 48 h at 37 °C. At these time points, cells were stained with Hoe in order to analyze only nucleated cells, with AxV and PI for detection of apoptotic and necrotic cells, and additionally with DiI for integrity of mitochondrial membrane potential as control for AxV/PI gating. The results of the viability staining with AxV and PI for one representative donor are shown in Figure 1.

As previously observed for the murine T cell line EL4, SPION^Citrate^ particle loading influenced the viability of primary human T cells as well [26]. Starting with an average cell viability of 94.4% ± 0.9% for all three donors, the loading of the T cells for 24 h decreased the proportion of viable cells from 90.2% ± 1.6% (control cells) to 75.9% ± 3.5% at an iron concentration of 100 µg/mL. This was accompanied by an increase of the proportion of apoptotic cells. With rising SPION^Citrate^ concentration and with prolonged incubation time, the proportion of apoptotic cells increased but no primary necrosis was to be detected due to the low toxicity of SPION^Citrate^. After 48 h, T cell viability dropped to 52.1% ± 2.8% at the highest particle concentration tested (100 µg Fe/mL). With longer observation time, we finally expect loss of plasma membrane integrity of initially apoptotic cells and the occurrence of secondary necrosis. We conclude that a prolonged incubation for 48 h of the T cells in the presence of high nanoparticle concentrations was too stressful for the cells. Therefore, the nanoparticle loading time for cell activation experiments was limited to 24 h.

### 3.3. Cellular Content of SPION^Citrate^

To be able to control SPION-loaded T cells with a magnetic field, the T cells must have a sufficiently high cellular SPION content. The magnetizability of the cells is then not dependent on whether the particles are internalized into the cell or adhere to the cell surface. To investigate the enrichment of SPION^Citrate^ in and/or on primary human T cells, three different methods were applied.

First, the change in the side scatter properties of the cells was investigated by flow cytometry. If the cells have interacted with SPION^Citrate^, either by uptake or adhesion, their granularity increased [31]. Since cellular morphology is altered by cell death processes as well, for the evaluation of SPION uptake by side scatter increase, it is necessary to gate on viable cells with intact mitochondrial membrane potential and exclude dying and dead cells from analysis.

As shown in Figure 2a, already, shortly after the addition of SPION^Citrate^ to the T cells, a significant increase in cell granularity was detected at iron concentrations ≥ 75 µg/mL. After incubation for 24 and 48 h, the SSc and therefore the cell granularity was even higher and increased also at lower concentrations, so that uptake and/or adhesion of the nanoparticles to the cells could be assumed.

For the differentiation between uptake and adhesion, the T cells were incubated with SPION^Citrate^ (25–100 µg Fe/mL) in the presence of the fluorescent dye Lucifer Yellow for 0, 24 and 48 h. Since LY is not membrane permeable itself, it cannot simply diffuse into the cells but must be actively incorporated, e.g., during particle uptake [34,35]. Thus, the internalization of SPION^Citrate^ in T cells can be estimated by flow cytometry based on the increasing LY fluorescence [24,26]. Here, it is mandatory to compare the treatment groups with the control of the corresponding point of time. This is because a time-dependent increase in fluorescence also occurs with the control cells due to ubiquitous endocytosis and thus, the uptake of LY into the cell [36]. As can be seen in Figure 2b, LY fluorescence of nanoparticle treated cells only increased significantly compared to the control cells after 48 h of incubation. This suggests that internalization of SPION^Citrate^ in primary human T cells can occur after prolonged incubation, but at earlier time points, we suspect that SPION^Citrate^ mainly adhere to the cell surface, particularly as T cells per se are described to typically have low phagocytic activity [37]. This is in line with findings by others who performed transmission electron microscopy analysis of T cells or NK cells incubated with SPIONs for two hours, showing that particles independently of their coating mainly adhered to the plasma membrane [23,25].

For quantifying the cellular content of SPION^Citrate^, T cells were incubated with particles for 24 h. SPION concentrations of 75 and 100 µg Fe/mL were selected based on toxicity, SSc and LY data. After incubation the cells were purified from excess nanoparticles, the cell count was determined, and the iron content of the cell pellet was measured by AES. For an iron concentration of 75 µg/mL, an average cell load of 0.09 pg ± 0.01 pg iron, as shown in Figure 2c, was achieved. The difference in loading between 75 µg Fe/mL and 100 µg Fe/mL was not statistically significant. We suggest that saturation of the cell surface with SPIONs occurred and therefore increasing particle concentrations did not lead to a higher loading. This saturation effect cannot be seen in SSc data since cells were not washed before flow cytometry analysis and therefore loosely bound SPIONs might be responsible for the dose-dependent SSc increase. Compared to the control cells (0.03 pg ± 0.03 pg Fe per cell), the cellular iron content was increased by loading with SPION^Citrate^.

However, compared to the already published values of approximately 1.0 pg Fe per cell achieved with the murine T cell line EL4, the loading was quite low [26]. For this, two reasons are postulated. On the one hand, the primary human T cells with a size of 8 µm as described by Renner et al. are much smaller than the murine EL4 cell line cells with 11 µm, as described by Ueda et al. [38,39]. Using these values, the volume of an EL4 cell is approximately 2.6 times higher than a human T cell’s volume. Additionally, the surface of an EL4 cell is about 1.89 times bigger. This was also reflected in the iron content of non-loaded T cells, since EL4 cells displayed an average iron content of 0.12 pg Fe per cell. On the other hand, and in contrast to the immortalized cell line, which triples within 24 h, the primary T cells do not proliferate without external stimuli. Therefore, the metabolic turnover as well as the “baseline” endocytosis of the primary human T cells is lower compared to the murine T cell line, which might partially explain the observed difference.

### 3.4. Magnetically Controllable T Cells

Despite the lower cellular iron content after loading with SPION^Citrate^ compared to our previous results, the possibility of controlling the loaded T cells by a magnetic field was tested. The cells were loaded with SPION^Citrate^ at an iron concentration of 75 µg/mL for 24 h, as described. Subsequently, they were purified from excess SPIONs and seeded into one chamber of a two-well chamber glass slide for their observation under the microscope. After focusing on the bottom plane at the edge of the separating wall of the two wells, a longitudinally magnetized block magnet was placed directly to the separating wall in the unused chamber and the cells’ movement was recorded. The magnetic flux density at the separating wall on the side of the cells was measured with a Hall effect magnetometer (FM302 with AS-NTM, Projekt Elektronik Mess- und Regelungstechnik, Berlin, Germany) and was about 240 mT. Supplementary Video S1 shows the behavior of the loaded cells without the magnet demonstrating no directed movements. Supplementary Video S2 shows the directed cell movement after the addition of the magnet to the right side of the chamber. These videos demonstrate the magnetizability of our SPION^Citrate^-loaded primary T cells.

There are a number of reasons why not all cells can be attracted equally well and equally fast. To be able to film as many cells as possible at the same time, the surface of the glass slide was chosen as plane. Due to sedimentation, most cells could be seen here, but as a result of contact with the surface, additional adhesion and friction forces had to be overcome before a cell could move in the direction of the magnet. Furthermore, it was not possible to load the cells homogeneously with SPION^Citrate^, as could be seen from the SSc distribution peaks. This might have resulted in faster attraction of cells with a higher particle content. In addition, the magnetic field strength weakens exponentially with increasing distance. Due to this effect, the most closely located cells experienced higher forces than those located further away.

As previously demonstrated for the murine T cell line EL4 and variously coated SPIONs, we have now also successfully loaded primary human T cells with SPION^Citrate^, allowing for their directed attraction by a magnetic field while maintaining a good cell viability.

However, before starting experiments in animal models it is necessary to test how the SPION-loaded T cells behave under flow conditions and at a greater distance from the magnet. For this purpose, a setup like the one used by Hennig et al. can be used [40]. Others also showed the ability of SPION-loaded T and NK cells to be magnetically attracted in a flow chamber in vitro [23,25]. The magnetic field of the electromagnet then used should also be modified to be as strong as possible and reach sufficiently deep into the tissue, whereby tumors close to the surface are of particular interest. Although high arterial blood pressure is actually detrimental to the accumulation of SPION-loaded T cells, it should not have a negative effect inside of the tumor since the vascular structure of a tumor is abnormal [41,42]. The vessels are more permeable, irregular and less organized, making it easier to accumulate T cells in the tumor than in areas with normal vascular structure. For this reason, we consider it possible that the magnetizability of T cells loaded with SPION^Citrate^ in a strong magnetic field is sufficient to enrich the cells in the tumor.

### 3.5. Effects of SPION^Citrate^ on T Cell Activation

In contrast to constantly dividing cell lines, primary T cells require a stimulus for their activation and proliferation. For this purpose, we used the ImmunoCult human CD3/CD28/CD2 T cell activator from Stemcell Technologies and also supplemented 30 IU/mL of rh IL-2. The use of CD2 was chosen because of its synergistic effect, which among other things reduces activation-induced cell death caused by CD3-triggered stimulation [43,44,45,46].

The effect of loading the T cells with SPION^Citrate^ on their ability to proliferate was investigated by three different methods: determination of the cell cycle status, live-cell imaging and CFSE dilution. For this purpose, T cells freshly isolated from human whole blood were loaded for 24 h with SPION^Citrate^ at an iron concentration of 75 µg/mL and purified from excess SPIONs.

**Figure 3:**
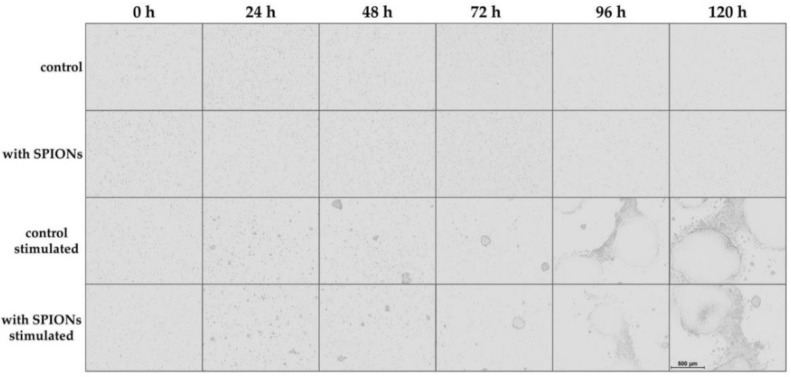
Live-cell imaging of proliferating T cells. Cells were incubated with citrate-coated superparamagnetic iron oxide nanoparticles (SPION^Citrate^) at an iron concentration of 75 µg/mL for 24 h. After purification, they were stimulated with CD3/CD28/CD2 activator mix and recombinant human interleukin-2. Live-cell imaging was performed every 24 h. The scale bar displays 500 µm. Abbreviations: SPIONs: superparamagnetic iron oxide nanoparticles, SPION^Citrate^: citrate-coated superparamagnetic iron oxide nanoparticles.

For live-cell imaging, we used the IncuCyte S3 Live-Cell Analysis System to observe the T cell proliferation by taking a phase contrast image every 24 h. By comparison of unstimulated and stimulated cells, their activation could be clearly seen, as displayed in Figure 3: initially, the T cells were equally distributed in the culture. After 24 h, the first small cell clusters were formed, and their size further increased over time. There was no obvious difference between non-loaded primary T cells and T cells loaded with SPION^Citrate^.

**Figure 4:**
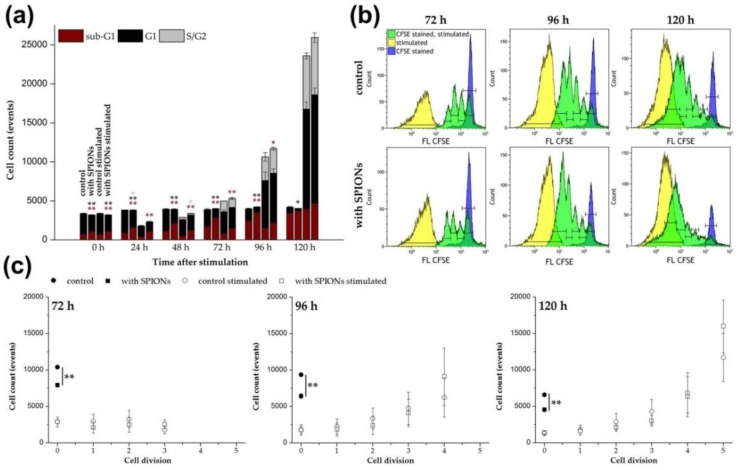
Impact of citrate-coated superparamagnetic iron oxide nanoparticles (SPION^Citrate^) on the proliferation of human primary T cells. Cells were loaded with SPION^Citrate^ at an iron concentration of 75 µg/mL for 24 h. After purification, they were stimulated with CD3/CD28/CD2 activator mix and recombinant human interleukin-2. (**a**) DNA content of cells was measured by lysing the T cells with Triton X-100 and staining with propidium iodide (PI) followed by flow cytometry evaluation. Cells in sub-G1 phase are displayed in red, in G1 phase in black and in S/G2 phase in grey. (**b**) Staining with carboxyfluorescein succinimidyl ester (CFSE) before stimulation was performed to allow for the analysis of T cell proliferation after stimulation by flow cytometry. CFSE stained, unstimulated T cells are shown in blue, whereas stimulated T cells are shown in yellow (no CFSE staining) and green (CFSE stained), respectively. Each green division peak represents one cell generation. (**c**) Count of cells in each generation after stimulation in (b) are displayed. The experiments were performed with three different donors. In (a) and (c), the mean values with standard deviations of one representative donor are shown. Data of other donors can be found in Supplementary Figure S4. Significance between loaded cells and control at the respective points of time is indicated by asterisks: * *p* < 0.05, ** *p* < 0.005. Abbreviations: CFSE: carboxyfluorescein succinimidyl ester, FL: fluorescence intensity, PI: propidium iodide, SPION^Citrate^: citrate-coated superparamagnetic iron oxide nanoparticles.

In addition to the visual evaluation of the cell proliferation, the cell cycle status of the cells was determined every 24 h after stimulation by flow cytometry using propidium iodide staining, as published by Riccardi et al. [33,47]. As shown in Figure 4a, the total cell number increased analogously to the live-cell imaging data. In unstimulated cells, the cell count was constant, but the proportion of T cells in the sub-G1 cell cycle phase increased from about 20% to up to 80% in non-loaded cells, reflecting cell death [47,48]. Loading with SPION^Citrate^ resulted in approximately 10% more cells in the sub-G1 phase. Stimulated cells behaved similar to unstimulated cells up to 48 h after stimulation. We assume that the initial decrease in cell count after stimulation was caused by the formation of proliferation clusters. The generation of single cell suspensions from these clusters was inefficient, which was also indicated by the presence of larger cell aggregates in the forward scatter/side scatter plot. However, beginning at 48 h, the cell numbers began to increase. Additionally, the proportion of T cells in the sub-G1 phase of both, loaded and non-loaded cells, decreased to less than 20%. In contrast, the percentage of dividing cells (S/G2 phase) increased to 20–30%.

The fluorescent dye CFSE can be used to monitor the proliferation of stimulated T cells by flow cytometry [49,50]. According to a protocol published by Quah et al., the cells were stained with CFDA SE prior to stimulation [32]. CFDA SE was used as the two acetate groups make it, in contrast to CFSE, well cell permeable and non-fluorescent [51]. The acetate groups of CFDA SE were cleaved by intracellular esterases and thereby, the fluorescent, non-cell permeable CFSE was formed. During each cell division, this dye was evenly distributed between the two daughter cells leading to a reduction of their CFSE fluorescence intensity to 50% with each cell division. In this way the cell proliferation could be tracked and the flow cytometry data for 72 to 120 h after stimulation are shown in Figure 4b. Each green peak represents a cell generation of the proliferating T cells. CFSE stained but unstimulated cells are shown in blue. They represent the native unstimulated cell population, which is referred to as cell generation 0 in Figure 4c. The yellow peaks indicate the autofluorescence of unstained stimulated cells. Figure 4c illustrates the exponential character of the T cell proliferation after stimulation by quantification of the cells in the different generations. Loading of the T cells with SPION^Citrate^ did not impair their proliferation. In contrast to unstimulated T cells where the nanoparticles induced a major decrease in cell numbers compared to the control over time, no difference was detectable after T cell stimulation. This observation is in line with the results obtained from the cell cycle analysis.

**Figure 5:**
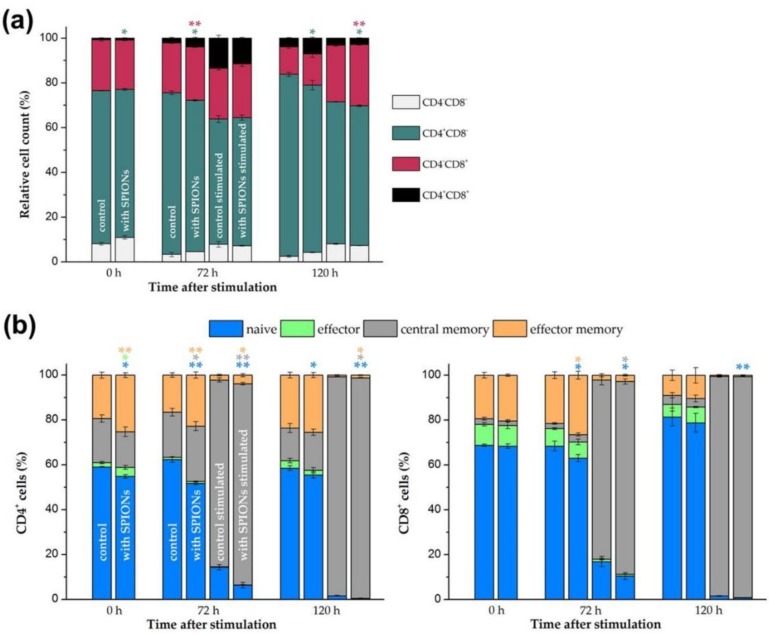
Phenotyping of T cell subsets. Primary human T cells were loaded with citrate-coated superparamagnetic iron oxide nanoparticles (SPION^Citrate^) at an iron concentration of 75 µg/mL for 24 h. After purification, they were stimulated with CD3/CD28/CD2 activator mix and recombinant human interleukin-2. (**a**) After 0, 72 and 120 h, T cells were stained with anti-CD4 and anti-CD8 antibodies and analyzed in flow cytometry to detect changes within CD4/CD8 ratio due to loading with SPION^Citrate^. (**b**) Cells were additionally stained with anti-CD197 (C-C motif chemokine receptor 7 (CCR7)) and anti-CD45RO antibodies for phenotyping of T cell subsets: naive (CCR7^+^CD45RO^−^), effector (CCR7^−^CD45RO^−^), effector memory (CCR7^−^CD45RO^+^) and central memory (CCR7^+^CD45RO^+^) T cells were identified by flow cytometry. The experiments were performed with three different donors. The mean values with standard deviations of one representative donor are shown. Data of other donors can be found in Supplementary Figure S5. Significance between loaded cells and control at the respective points of time is indicated by asterisks: * *p* < 0.05, ** *p* < 0.005. Abbreviations: CCR7: C-C motif chemokine receptor 7, SPION^Citrate^: citrate-coated superparamagnetic iron oxide nanoparticles.

As already shown after isolation of T cells from whole blood in Supplementary Figure S1, the isolation of the cells did not have a major effect on the proportion of CD4^+^ helper and CD8^+^ cytotoxic T cells. This was also checked during the stimulation in the presence of nanoparticles. After isolation, loading and purification, the T cells were stimulated, and after 0, 72 and 120 h, stained with anti-CD4 and anti-CD8 antibodies before measurement by flow cytometry. As shown in Figure 5a, a comparison of the stimulated loaded cells with the stimulated control cells showed only a slight shift in the CD4/CD8 distribution in favor of the CD8^+^ cells after 120 h in case of nanoparticle loading.

In addition, the T cells were stained with anti-CD197 (CCR7) and anti-CD45RO antibodies to phenotype different T cell subpopulations. Naive (CCR7^+^CD45RO^−^), effector (CCR7^−^CD45RO^−^), effector memory (CCR7^−^CD45RO^+^) and central memory (CCR7^+^CD45RO^+^) T cells could thus be identified for CD4^+^ and CD8^+^ T cells, respectively [52,53]. As shown in Figure 5b, the polyclonal T cell stimulation reduced the number of naive, effector and effector memory CD4^+^ as well as CD8^+^ T cells and increased the proportion of central memory T cells. Of note, the loading of the primary human T cells with the SPION^Citrate^ particles enhanced this effect. T cell differentiation and function has been described to be accompanied by alterations in cell metabolism [54]. Interestingly, higher rates of mitochondrial respiration and glycolysis have been detected by others in magnetic nanoparticle-treated primary murine T cells, stating that alteration of the T cell metabolism by nanoparticles might be a sign for transition to an activated T cell state [25].

**Figure 6:**
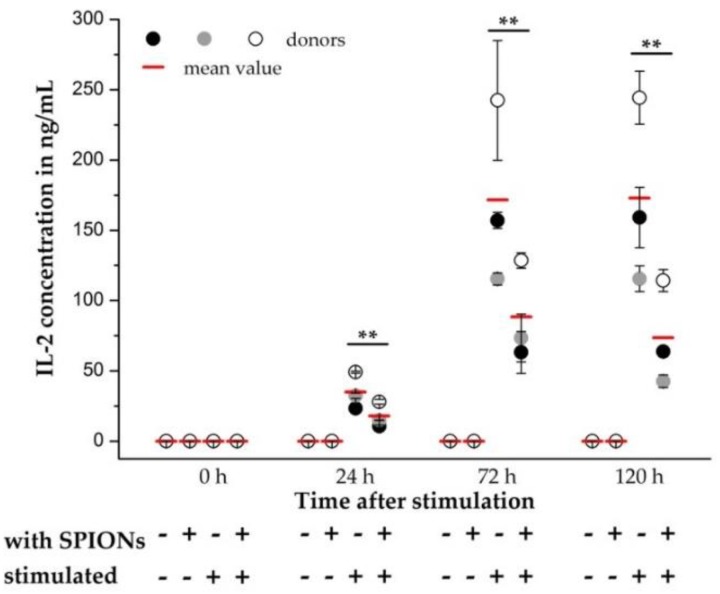
Quantification of interleukin-2 (IL-2) release by primary human T cells. T cells were loaded with citrate-coated superparamagnetic iron oxide nanoparticles (SPION^Citrate^) at an iron concentration of 75 µg/mL for 24 h. After purification, they were stimulated with CD3/CD28/CD2 activator mix. The addition of recombinant human IL-2 was omitted. 0, 24, 72 and 120 h after stimulation, supernatants were collected and the amount of secreted IL-2 was measured by sandwich enzyme-linked immunosorbent assay (ELISA). The experiment was performed with three different donors. The mean values with standard deviations of each of the donors are shown as circles, their overall mean value as a red line. Significance between the overall mean values of loaded cells and control at the respective points of time is indicated by asterisks: ** *p* < 0.005. Abbreviations: ELISA: enzyme-linked immunosorbent assay, IL-2: interleukin-2, SPION^Citrate^: citrate-coated superparamagnetic iron oxide nanoparticles.

As a further parameter for the influence of SPION^Citrate^ on the stimulation of T cells, the release of IL-2 from the primary human T cells was quantified. In this case, the addition of rh IL-2 for stimulation of T cells was omitted and the secreted amount of IL-2 in the supernatant was quantified by sandwich enzyme-linked immunosorbent assay (ELISA) at 0, 24, 72 and 120 h after stimulation. In Figure 6, the mean values of three different donors were compared and a significant decrease in IL-2 release was found. Similar observations have already been made by Yan et al., who showed a reduced IL-2 production of the human T cell line Jurkat (clone E6-1) after incubation with their SPIONs [55]. Yan et al. even postulated that the reduced expression of inflammatory cytokines such as IL-2 might be responsible for the low cytotoxicity of the particles. Although the secretion of IL-2 was reduced by loading the T cells with SPION^Citrate^, the expression of CD25, which is part of the IL-2 receptor, was not altered. Since in the simultaneously performed live-cell imaging no difference between the proliferation of T cells with and without rh IL-2 supplementation was recognizable, we assume that the reduced amount of released IL-2 was still sufficient for the autocrine stimulation of T cell proliferation.

**Figure 7:**
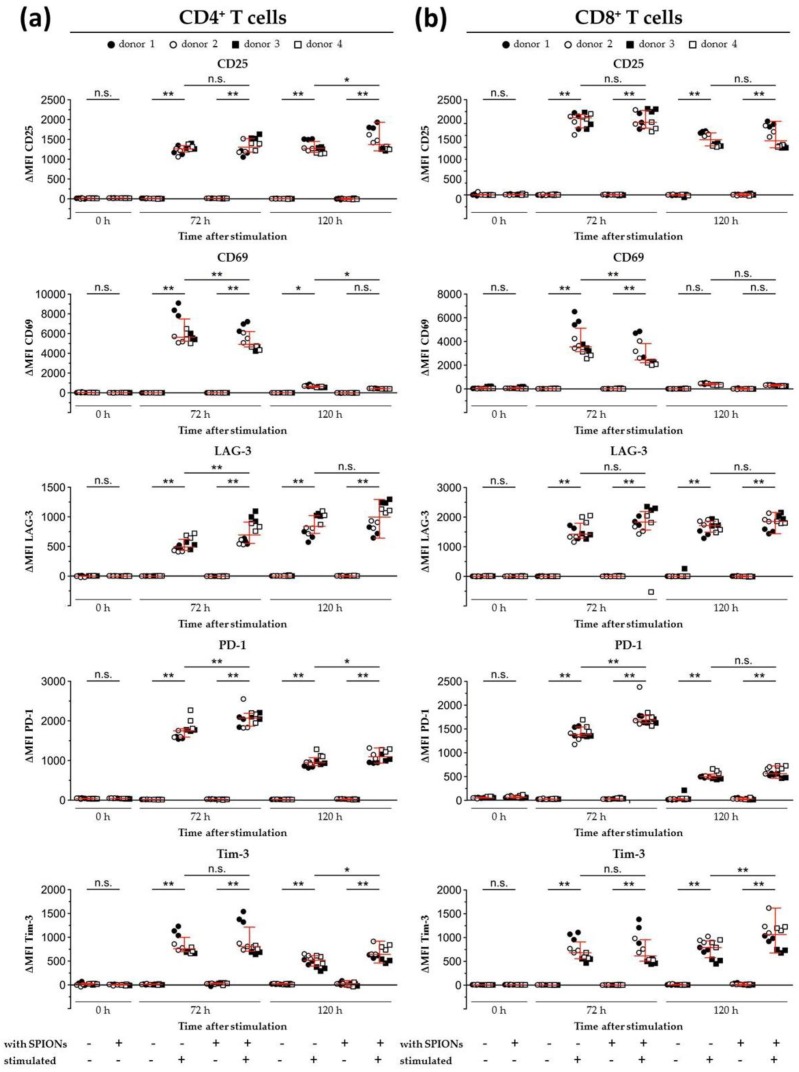
Expression of activation markers and inhibitory cell surface molecules on isolated primary humanT lymphocytes. T cells were loaded with citrate-coated superparamagnetic iron oxide nanoparticles (SPION^Citrate^) at an iron concentration of 75 µg/mL for 24 h. After purification, they were stimulated with CD3/CD28/CD2 activator mix and recombinant human interleukin-2. 0, 72 and 120 h after stimulation, T cells were stained with anti-CD4 and anti-CD8 antibodies as well as antibodies against the inhibitory cell surface molecules programmed cell death 1 (PD-1), lymphocyte activation gene 3 (LAG-3), T cell immunoglobulin and mucin domain containing 3 (Tim-3), and the T cell activation markers CD25 and CD69 and analyzed in flow cytometry. Results for CD4^+^ T cells are displayed in (**a**) and CD8^+^ T cells in (**b**). The experiments were performed with four different donors. Significance between the mean values of all donors of loaded cells and control at the respective points of time is indicated as follows: not significant (n.s.), *p* > 0.05, * *p* < 0.05, ** *p* < 0.005. Abbreviations: LAG-3: lymphocyte activation gene 3, MFI: median fluorescence intensity, PD-1: programmed cell death 1, SPION^Citrate^: citrate-coated superparamagnetic iron oxide nanoparticles, Tim-3: T cell immunoglobulin and mucin domain containing 3.

In addition to analyzing the influence of loading the T cells with SPION^Citrate^ on the release of IL-2, the expression of different activation markers was also investigated. Cell surface staining for the activation markers CD25 and CD69 as well as the checkpoint molecules programmed cell death 1 (PD-1, CD279), lymphocyte activation gene 3 (LAG-3, CD223), and T cell immunoglobulin and mucin domain containing 3 (Tim-3, CD366) were analyzed by flow cytometry [56,57,58,59,60,61,62,63,64]. After exclusion of doublets and higher cell aggregates, dead cells were excluded by DAPI staining and only DAPI^−^ cells were evaluated [65]. As shown in Figure 7, SPION^Citrate^-loaded primary human T cells were compared to non-loaded cells after 72 and 120 h of stimulation with CD3/CD28/CD2 activator mix and rh IL-2. Both SPION^Citrate^-loaded primary human CD4^+^ and CD8^+^ T cells displayed a comparable CD25 cell surface expression after stimulation. For the early point of time (72 h), SPION^Citrate^-loaded primary human CD4^+^ as well as CD8^+^ T cells displayed slightly less CD69 and slightly more LAG-3 and PD-1. However, after 120 h of stimulation, no or only subtle differences in the cell surface expression of CD69, LAG-3 and PD-1 between SPION^Citrate^-loaded and non-loaded T cells could be observed. Both CD4^+^ and CD8^+^ T cells showed a higher cell surface expression of Tim-3 after 120 h of stimulation. These data are in line with findings by others who showed that cell surface markers on Jurkat cells, primary murine T cells as well as on antigen-specific OT-1 CD8^+^ T cells were not altered by magnetic nanoparticle loading [25,66]. Taken together, our results demonstrate that SPION^Citrate^ had only a minor effect on the stimulatory capacity of primary human T cells and might even support the differentiation of naive into central memory T cells.

## 4. Conclusions

So far, primary T cells have rarely been used for studying the uptake of superparamagnetic nanoparticles and thus, it was not clear if nanoparticle loading impacts effector functions of these cells. In this study, we succeeded in loading freshly isolated primary human CD3^+^ T cells with citrate-coated superparamagnetic iron oxide nanoparticles. The loaded cells could be attracted by magnetic forces while maintaining high cell viability. Furthermore, the functionality of the SPION^Citrate^-loaded T cells was tested in comparison to non-loaded T cells in order to ensure that the primary cells were not impaired in their immunological responses by the SPION-loading, and thus retained their full functionality for the purpose of tumor treatment. Besides a lower IL-2 secretion after loading with SPION^Citrate^, the particles had only minor effects on the T cell stimulation and proliferation.

In future studies, we aim to investigate antigen-specific stimulation and tumor cell killing by SPION^Citrate^-loaded primary human T cells as well as their behavior under flow conditions. Additionally, we will transfer our knowledge to primary murine T cells to allow for in vivo testing of T cell functionality and magnetizability within tumor bearing mice.

## Figures and Tables

**Table 1:** T cell phenotyping and activation markers.

Fluorescence	Antibody	Clone	Isotype Control	Clone
FITC	anti-human CD3	UCHT1	mouse IgG1, κ	MOPC-21
PerCP-Cy5.5	anti-human CD4	OKT4	mouse IgG2b, κ	MPC-11
Pacific Blue	anti-human CD8	HIT8a	mouse IgG1, κ	MOPC-21
PE	anti-human CD197 (CCR7)	G043H7	mouse IgG2a, κ	MOPC-173
APC	anti-human CD45RO	UCHL1	mouse IgG2a, κ	MOPC-173

Abbreviations: APC: allophycocyanin, CCR7: C-C motif chemokine receptor 7, FITC: fluorescein isothiocyanate, IgG: immunoglobulin G, PE: phycoerythrin, PerCP-Cy5.5: peridinin-chlorophyll-protein complex-cyanine 5.5.

**Table 2:** T cell phenotyping and activation markers.

Fluorescence	Antibody	Clone	Isotype Control	Clone
BUV395	anti-human CD3	UCHT1	-	
PE	anti-human CD4	RPA-T4	-	
BUV 737	anti-human CD8	SK1	-	
BV 650	anti-human CD279 (PD-1)	EH12.2H7	mouse IgG1, κ	MOPC-21
BV 711	anti-human CD223 (LAG-3)	11C3C65	mouse IgG1, κ	MOPC-21
PE/Dazzle594	anti-human CD366 (Tim-3)	F38-2E2	mouse IgG1, κ	MOPC-21
PerCP-Cy5.5	anti-human CD25	BC96	mouse IgG1, κ	P3.6.2.8.1
PE-Cy7	anti-human CD69	FN50	mouse IgG1, κ	MOPC-21

Abbreviations: BUV: Brilliant Ultraviolet, BV: Brilliant Violet, Cy: cyanine, IgG: immunoglobulin G, LAG-3: lymphocyte activation gene 3, PD-1: programmed cell death 1, PE: phycoerythrin, PerCP: peridinin-chlorophyll-protein complex, Tim-3: T cell immunoglobulin and mucin domain containing 3.

## Supplementary Materials

The following are available online at https://www.mdpi.com/2073-4409/9/2/342/s1, Figures S2–S5 show additional data of two more donors to Figure 1, Figure 2, Figure 4 and Figure 5. Figure S1: Isolation of CD3^+^ T cells from human whole blood. Figure S2: Effects of SPION^Citrate^ on viability of human primary T lymphocytes. Figure S3: Uptake of SPION^Citrate^ into human primary T lymphocytes. Figure S4: Impact of SPION^Citrate^ on the proliferation of isolated human primary T cells. Figure S5: Phenotyping of T cell subsets. Table S1. Distribution of CD4^+^ and CD8^+^ T cells before and after isolation. Video S1: T cells loaded with SPION^Citrate^ in one well of a two-chamber glass slide did not show targeted movement in absence of a magnet. Video S2: T cells loaded with SPION^Citrate^ in the left well of a two-chamber glass slide showed targeted movement in the direction of a magnet which was placed in the well to the right.
