# Supplementary material for: Loading of Primary Human T Lymphocytes with Citrate-Coated Superparamagnetic Iron Oxide Nanoparticles Does Not Impair Their Activation after Polyclonal Stimulation
Source: Cells. 2020 Feb 1;9(2):342. doi: 10.3390/cells9020342 (PMC7072432; doi:10.3390/cells9020342)
Supplement: Supplementary file 1 [file cells-09-00342-s001.pdf]

cells

MDPI

Supplementary Materials

Loading of Primary Human T Lymphocytes with Citrate-Coated Superparamagnetic Iron Oxide Nanoparticles Does Not Impair Their Activation after Polyclonal Stimulation

Marina Mühlberger, Harald Unterweger, Julia Band, Christian Lehmann, Lukas Heger, Diana Dudziak, Christoph Alexiou, Geoffrey Lee and Christina Janko

Supplementary Results:

$CD3^+$ T cells can be enriched directly from whole blood without changing the proportion of $CD4^+$ and $CD8^+$ T cells

To investigate the feasibility of magnetic labelling of primary T lymphocytes from human whole blood, $CD3^+$ T cells were positively enriched using the Fab TACS Gravity Kit from IBA. As described by Weiss et al, this method immobilizes Strep-tagged Fab fragments of an anti-CD3 antibody on a Strep-Tactin-coated agarose column [1].

In order to investigate whether there was a change in the ratio between $CD3^+CD4^+$ and $CD3^+CD8^+$ cells, both whole blood samples after lysis of the erythrocytes and isolated cells were stained with anti-CD3, anti-CD4 and anti-CD8 antibodies and the distribution of the immune cell subsets was measured by flow cytometry. Leukocyte populations were discriminated into granulocytes, monocytes and lymphocytes by their morphological properties regarding size and granularity by analyzing forward (FSc) and side scatter (SSc) as shown in **Figure S1**. **Table S1** displays that the cell isolation from blood did not have a major effect on the proportion of $CD4^+$ and $CD8^+$ T cells.

1. Weiss, R.; Gerdes, W.; Leonhardt, F.; Berthold, R.; Sack, U.; Grahnert, A. A comparative study of two separation methods to isolate monocytes. *Cytometry. Part A : the journal of the International Society for Analytical Cytology* **2019**, *95*, 234-241, doi:10.1002/cyto.a.23633.

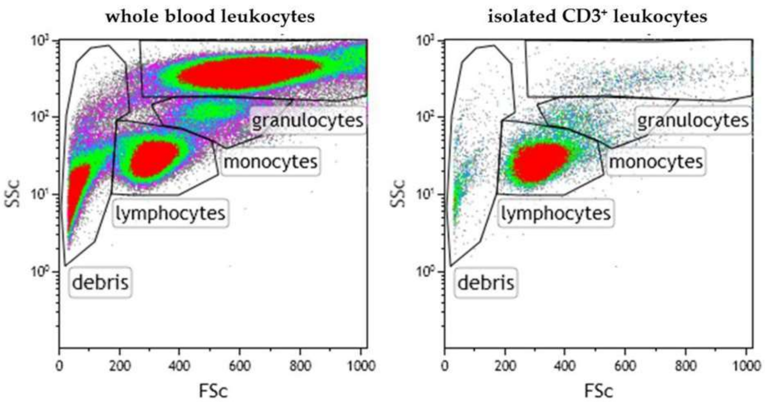

	whole blood	isolated
lymphocytes	17%	90%
→ CD4⁺	→ 69%	→ 66%
→ CD8⁺	→ 27%	→ 30%
monocytes	3%	4%
granulocytes	68%	2%
debris	12%	3%

Figure S1. Isolation of $CD3^+$ T cells from human whole blood. Upper row left: human whole blood before T cell isolation, right: isolated T cells. Lower row: percentages of cell populations in whole blood and isolated T cell fraction. . The experiment was performed with three different donors. Shown are the results of one representative donor.

Abbreviations: FSc: forward scatter, SSc: side scatter.

Table 1. Distribution of $CD4^+$ and $CD8^+$ T cells before and after isolation. The proportion of CD4+ and CD8+ T cells is not altered by the isolation.

**Donor**	**$CD4^+$ T cells**			**$CD8^+$ T cells**		
	blood	isolated	difference	blood	isolated	difference
1	62.1%	55.7%	-6.4%	28.4%	32.1%	+3.7%
2	68.8%	66.1%	-2.7%	26.9%	29.7%	+2.8%
3	88.1%	87.7%	-0.4%	9.0%	10.6%	+1.6%
average			-3.2% ± 2.5%			+2.7% ± 0.9%

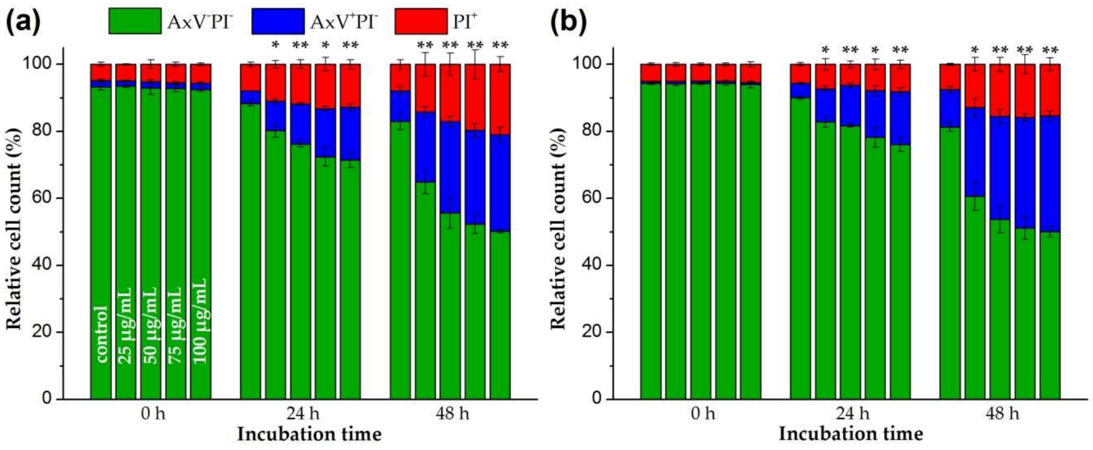

Figure 2. Effects of SPION^{Citrate} on viability of human primary T lymphocytes. T cells were incubated with SPION^{Citrate} at various iron concentrations (0 – 100 µg/mL). After 0, 24 and 48 hours, cells were stained with AxV and PI to detect apoptotic and necrotic cells by flow cytometry. Viable cells (AxV$^-$ PI$^-$) are displayed in green, apoptotic cells (AxV$^+$PI$^-$) in blue and necrotic cells (PI$^+$) in red. **(a)** displays donor 2, **(b)** donor 3. The experiment was performed with three different donors. Shown are the mean values with standard deviations. Significance for viable cells between treatment groups and control at the respective time is indicated by asterisks: (* $p < 0.05$, ** $p < 0.005$).

Abbreviations: AxV: Annexin A5 FITC conjugate, FITC: fluorescein isothiocyanate, PI: propidium iodide, SPION^{Citrate}: citrate-coated superparamagnetic iron oxide nanoparticles.

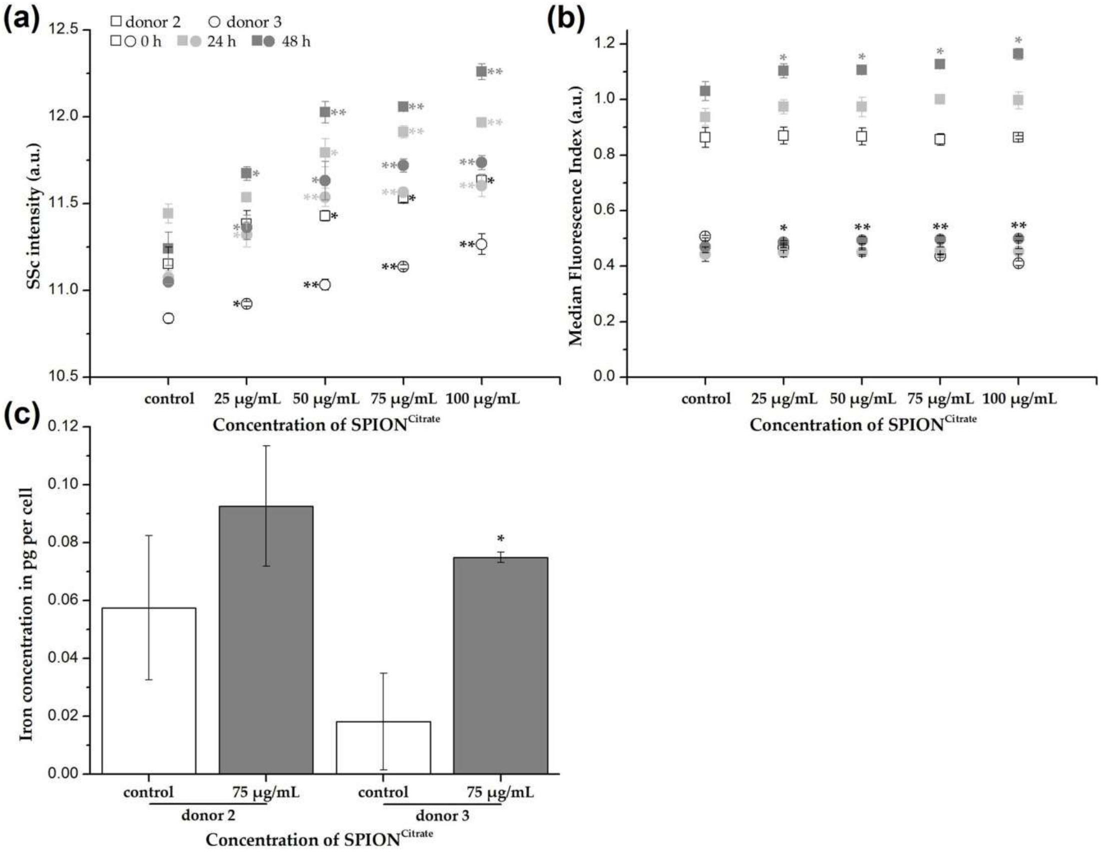

Figure 3. Uptake of SPION^{Citrate} into human primary T lymphocytes. **(a)** Changes in cellular granularity due to SPION uptake or adhesion were determined by measuring the side scatter (SSc) in

flow cytometry after incubation of T cells with $SPION^{Citrate}$. Since cell death processes alter cellular morphology as well, we gated on viable DiI^+ cells with intact mitochondrial membrane potential. **(b)** T cells were incubated for 0, 24 and 48 hours with $SPION^{Citrate}$ (25 – 100 µg Fe/mL) and the fluorescent dye Lucifer Yellow (LY). LY gets co-ingested with nanoparticles and served therefore as indicator for intracellular SPION uptake. Since LY leaks from disrupted membranes, we gated on viable DiI^+ cells with intact mitochondrial membrane potential. **(c)** Quantification of cellular iron content was performed by atomic emission spectroscopy after incubation of T cells with $SPION^{Citrate}$ (75 and 100 µg Fe/mL) for 24 hours. The experiments were performed with three different donors. Shown are the mean values with standard deviations. Significance between treatment groups and control at the respective time is indicated by asterisks: * $p < 0.05$, ** $p < 0.005$.

Abbreviations: DiI: $DiIC_1(5)$ (1,1′-dimethyl-3,3,3′,3′-tetramethylindodicarbocyanine iodide), LY: Lucifer Yellow, SPION: superparamagnetic iron oxide nanoparticle, $SPION^{Citrate}$: citrate-coated superparamagnetic iron oxide nanoparticles, SSc: side scatter

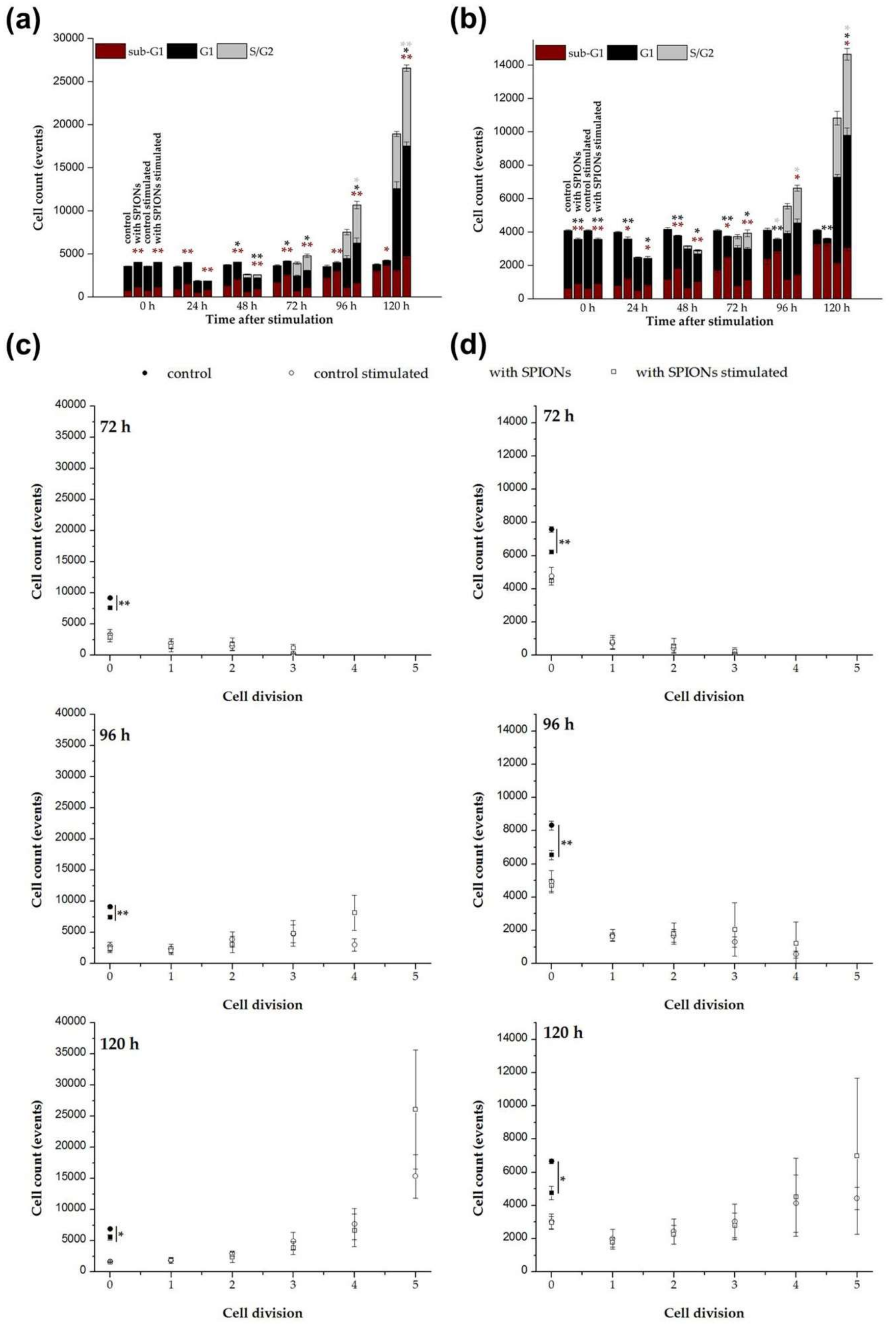

Figure 4. Impact of SPION^{Citrate} on the proliferation of isolated human primary T cells. Cells were loaded with SPION^{Citrate} at an iron concentration of 75 µg/mL for 24 hours. After purification, they were stimulated with CD3/CD28/CD2 activator mix and rh IL-2. **(a)** and **(b)** DNA content of cells was

measured by lyzing the T cells with Triton X-100 and staining with PI followed by flow cytometry evaluation. Cells in sub-G1 phase are displayed in red, in G1 phase in black and in S/G2 phase in grey. **(a)** displays donor 2, **(b)** donor 3. **(c)** and **(d)** Staining with CFSE before stimulation was performed to allow for the analysis of T cell proliferation after stimulation. Count of cells in each generation after stimulation are displayed. **(c)** displays donor 2, **(d)** donor 3. The experiments were performed with three different donors. The mean values with standard deviations are shown. Significance between loaded cells and control at the respective points of time is indicated by asterisks: * $p < 0.05$, ** $p < 0.005$.

Abbreviations: CFSE: carboxyfluorescein succinimidyl ester, PI: propidium iodide, rh IL-2: recombinant human interleukin-2, SPION^{Citrate}: citrate-coated superparamagnetic iron oxide nanoparticles

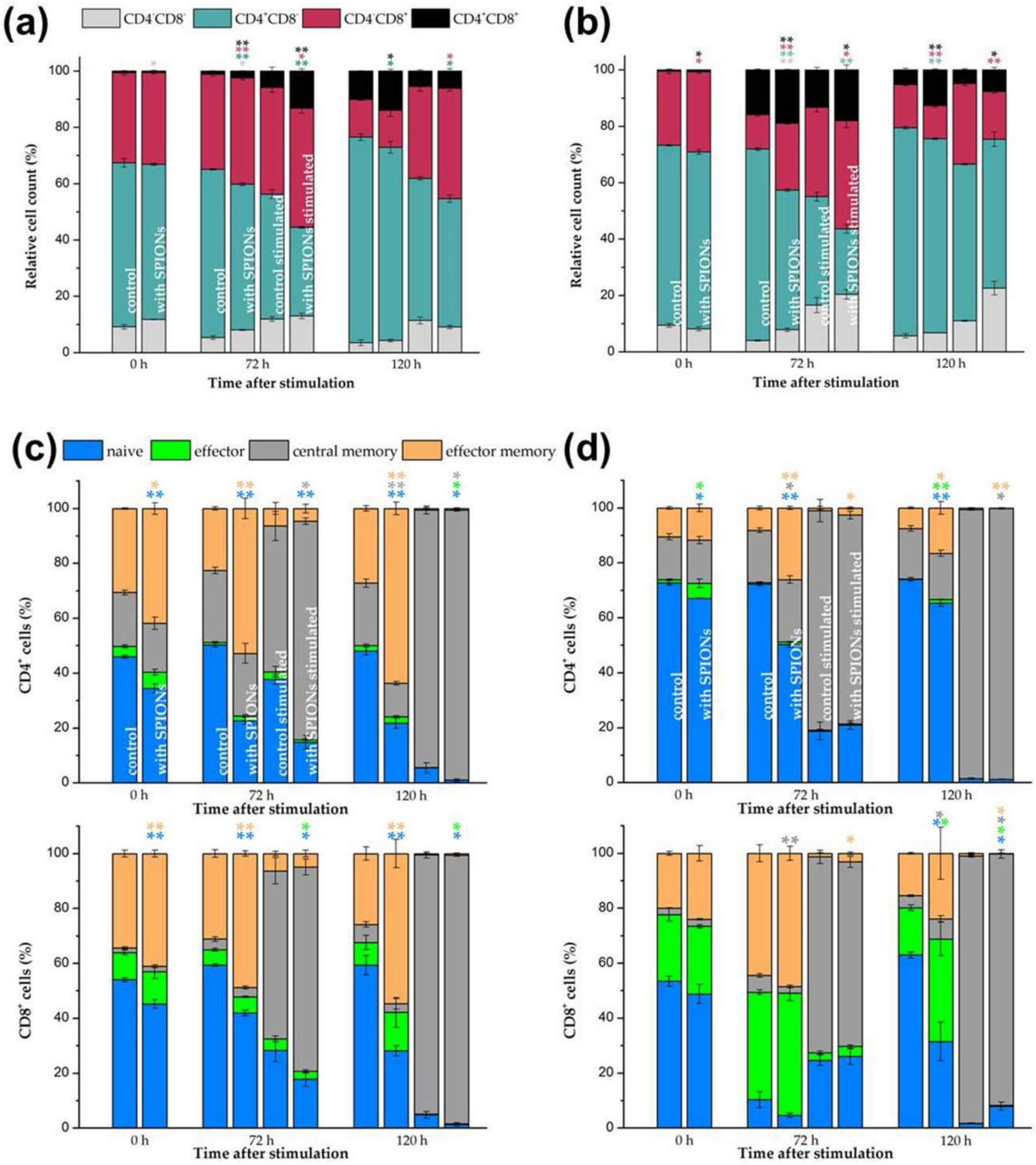

Figure 5. Phenotyping of T cell subsets. Primary human T cells were loaded with SPION^{Citrate} at an iron concentration of 75 µg/mL for 24 hours. After purification, they were stimulated with CD3/CD28/CD2 activator mix and rh IL-2. **(a)** and **(b)** After 0, 72 and 120 hours, T cells were stained with anti-CD4 and anti-CD8 antibodies to detect changes within CD4/CD8 ratio due to loading with

SPION^{Citrate}. **(a)** displays donor 2, **(b)** donor 3. **(c)** and **(d)** Cells were additionally stained with anti-CD197 (CCR7) and anti-CD45RO antibodies for phenotyping of T cell subsets: naive ($CCR7^{+}CD45RO^{-}$), effector ($CCR7^{-}CD45RO^{-}$), effector memory ($CCR7^{-}CD45RO^{+}$) and central memory ($CCR7^{+}CD45RO^{+}$) T cells were identified. **(c)** displays donor 2, **(d)** donor 3. The experiments were performed with three different donors. The mean values with standard deviations are shown. Significance between loaded cells and control at the respective points of time is indicated by asterisks: * $p < 0.05$, ** $p < 0.005$.

Abbreviations: CCR7: C-C motif chemokine receptor 7, rh IL-2: recombinant human interleukin-2, SPION^{Citrate}: citrate-coated superparamagnetic iron oxide nanoparticles
